# Photoacoustic signal-to-noise ratio comparison for pulse and continuous waveforms of very low optical fluence

**DOI:** 10.1117/1.JBO.27.7.076006

**Published:** 2022-07-26

**Authors:** DongYel Kang

**Affiliations:** Hanbat National University, School of Basic Sciences, Daejeon, Republic of Korea

**Keywords:** photoacoustics, photoacoustic imaging, signal-to-noise ratio, simulation, theoretical analysis

## Abstract

**Significance:**

A majority in the photoacoustic (PA) community unconditionally accepts that pulse PA signals show much higher signal-to-noise ratios (SNRs) than continuously excited PA signals. However, we indicate this existing notion would not be valid for very low optical-fluence light-emiting diodes (LEDs)/laser diodes (LDs)-based PA systems.

**Aim:**

We demonstrate in theory and simulation that when the optical fluence of PA-excitation waveforms is much lower than the American National Standards Institute (ANSI) maximum permission exposure (MPE), matched filtered PA signals from chirp waveforms show higher SNRs than those of pulse train waveforms.

**Approach:**

We theoretically derive the PA SNR expression considering the pulse fluence reduction factor based on the ANSI MPE. We investigate and analyze SNR ratios of the pulse train and chirp-waveform matched filtered PA signals with conceptual understanding. We also perform brute-force simulations to extract PA SNRs for the verification of the result.

**Results:**

The brute-force simulations show that the matched filtering with chirp waveforms could achieve better SNRs than pulse train waveforms for very low-fluence PA systems. As the fluence is smaller, the SNR of the matched filtered PA signals is more dominant than that of pulse trains in a wider PA data acquisition time range. In addition, estimated SNR ratios adopting actual parameters of LED/LD-based pulse train PA systems in previous literature support the finding of this paper.

**Conclusions:**

The result can extend the possibility of applying various continuous waveform techniques already studied in the conventional radar technology to PA systems of limited optical power, which would diversify and expedite the research and development of LED/LD-based, compact, and cost-effective PA systems.

## 1 Introduction

The mechanical expansion of photon-absorbing objects thermally excited by a temporally varying light source generates ultrasonic waves, known as the photoacoustic (PA) effect. For decades, various imaging modalities adapting this PA effect have been developed in the field of biomedical optics due to several distinctive advantages over purely optical imaging,^1^^–^^4^ such as a very low scattering degree in a biomedical tissue. Also, PA excitation by optical sources of several different wavelengths could extend PA applicability to a variety of biomedical optics sub-fields because of the capability of spectral analysis on a target absorbing object.^3^^,^^5^ Generally, the magnitude of induced PA signals is proportional to the absorbed photon energy that is mainly determined by the absorption coefficient of an absorbing object and radiant exposure (i.e., fluence) on it.^2^^,^^4^ The fluence of an incident optical beam on human skin is limited by the maximum permissible exposure (MPE) of the American National Standards Institute (ANSI)^6^ and is quickly attenuated inside a biomedical tissue. Therefore, the signal-to-noise ratio (SNR) of PA signals induced from biomedical subjects is typically very low with an inherently existing ultrasound thermal noise, which is one of the obstacles to the extension of biomedical fields utilizing the PA phenomenon.^7^^,^^8^

The optical sources for the generation of PA waves are categorized into pulse and continuous waveforms.^4^^,^^9^ For the pulse-mode PA system, tens of nanoseconds pulses are illuminated, where the depth information of absorbing objects can be straightforwardly obtained from the time of flight of induced pulse PA waves. The continuous-mode PA system uses continuously varying waveforms, which could have the greater versatility and applicability than the pulse-mode due to the room of modifying continuous optical waveforms demonstrated in the modern radar technology.^4^^,^^10^^,^^11^ However, it has been commonly accepted that the SNR of continuously induced PA signals is much lower than that of pulse ones.^12^^,^^13^ One of the attempts to compensate for the low SNR is to chirp the modulation frequency of an incident waveform in a MHz range, which is called PA radar.^10^^,^^13^ An PA radar signal generated by the chirp optical waveform is filtered by the cross-correlation with the original chirp waveform. This matched filtering process results in a compressed PA peak with improved SNRs, and the temporal position of this peak directly represents the depth of an absorbing object, similar to the time of flight in pulse PA imaging. However, even adopting the PA radar approach, it has been reported that the matched filtered PA signals show 20 to 40 dB lower SNRs than pulse PA signals.^9^^,^^12^^–^^14^ Because of the relatively high SNR, pulsed PA imaging has been the mainstream in the field of PA biomedical optics.

High-power tunable pulse lasers for PA spectroscopic imaging, such as optical parametric oscillator and dye lasers, are expensive and bulky. These drawbacks limit the suitability of pulse PA sources for constructing cost-effective, compact, and multi-wavelength PA imaging systems. In addition, high-power tunable pulse lasers typically have pulse repetition rates smaller than several tens of Hz, which implies the inadequacy of performing real-time PA measurements, such as monitoring rapidly changing biomedical properties.^4^^,^^15^ Recently, light-emitting diodes (LEDs) and laser diodes (LDs) have been extensively considered as PA excitation optical sources.^15^ Commercially available LEDs and LDs are compact and relatively inexpensive, which addresses the existing drawbacks inherent in bulky pulse lasers. Using multiple LEDs or LDs distributed in a wide wavelength range could enable spectroscopic PA measurements compactly and cost-effectively. Almost all commercially developed LEDs and LDs are inherently designed for a continuous light output. However, due to existing conventional thought that pulse PA signals exhibit higher SNRs than continuous ones, the majority of LED/LD-based PA imaging studies have been conducted with pulse-mode or pulse LDs.^14^^,^^16^^–^^18^ The usage of pulse LDs limits the development of PA spectroscopic imaging systems because the typical wavelength of most commercially available pulse LDs is in the range of near-infrared more than ∼800  nm.^15^^,^^17^^,^^18^ Additionally, most pulse LDs operate with a very low power per pulse, which could lead to the reduction of overall fluence and real-time PA measurement capability. For example, Hariri et al.^19^ implemented transmission-mode PA microscopy imaging with the pulse LD of a repetition rate up to 20 KHz, but the power per pulse was only in the range of tens of nJ. Due to this low power, they enhanced the PA SNR by averaging PA signals 200 to 5000 times, which would significantly reduce the PA measurement speed. As an alternative to overcome the limited wavelength availability of pulse LDs, it has been actively conducted to measure PA signals by operating continuous LEDs and LDs with pulse current drivers. Dai et al.^20^ imaged the vasculature of a mouse ear *in-vivo* using the customized LED driver that operated LEDs in the pulse-mode at 40-KHz repetition rate of 200-ns pulse width. Stylogiannis et al.^21^ realized fast PA imaging with overdriven LDs that fired 10-ns pulses at 625-KHz repetition rate. Especially, Zhong et al.^22^ constructed the fingertip-LD PA imaging system that was operated in both pulse and continuous modes.

In the previous studies comparing SNRs of PA signals by pulse and continuous optical waveforms, it was assumed that the fluence of those incident beams reached the ANSI MPE and the pulse width was less than several nanoseconds.^9^^,^^12^^–^^14^ Due to the very low optical power of commercially available compact LEDs or LDs, achieving the beam fluence up to the ANSI MPE with such a short pulse duration is almost impossible even though it partly depends on optical beam focusing capability of illumination optical systems. To compensate for the low output power of LEDs and LDs, methods of stacking several LED and LD components or extending the duration of pulse beams up to several hundred nanoseconds have been attempted.^18^^–^^22^ Although with these attempts, the fluence per pulse of LEDs/LDs used as PA sources is still hundreds to thousands of times lower than the ANSI MPE. Moreover, pulse incident beams of more than several tens of nanoseconds are not delta-like pulse waveforms assumed in the previous PA SNR studies, so it is necessary to conduct further analysis about the effect of such a long pulse duration on the SNR of pulse PA signals. Petschke and La Riviere^12^ concluded that the SNRs of PA signals by single and collective pulse waveforms (i.e., pulse train) are much higher than those of matched-filtered PA radar signals considering all waveforms had the same frequency bandwidth and duration. However, their result was under the assumption that the fluence of all waveforms reached to the ANSI MPE and the pulse width was short enough to consider the constant-magnitude spectrum within the measurement bandwidth. Also, despite of a limited repetition rate in most practical LED/LD-based pulse train systems, they assumed that the number of pulses in the pulse train could be maximized as long as the ANSI MPE is fulfilled, which implies the possibility of exaggerated SNRs for pulse train PA signals.

In this paper, we investigate the SNRs of PA signals induced by a single pulse, pulse train, and continuous chirp waveforms when the fluence of these optical beams is much lower than the ANSI MPE, as commonly happens in LED/LD-based PA imaging systems. If there is no absolute benefit on pulse PA signals in terms of an SNR, it is not necessary to stick to pulse-mode PA measurements with LEDs/LDs considering extra customized pulse current drivers. First, we derive the PA SNR expression in the frequency domain to analytically compare the SNRs for pulse and continuous waveforms. Next, by applying the reasonable assumptions to the frequency domain SNR expression, we derive the SNR ratio between PA signals from single pulse and matched filtering with a chirp waveform. Starting from this theoretically derived SNR ratio form, we investigate and analyze SNR ratios for pulse train and chirp-waveform matched filtered PA signals considering the same bandwidth and duration. Especially, we examine how the SNR ratios are characterized with the parameters of practically constructed LED/LD pulse-mode PA systems reported in the previous literature. Finally, we present the conclusion with discussion, which might significantly influence the research trend of constructing compact, cost-effective PA spectroscopic imaging systems.

## 2 Photoacoustic SNR in the Frequency-Domain

The overall process of deriving the PA SNR expression in the frequency domain follows the description in the previous literature.^23^^,^^24^ Consider a localized absorbing object bounded as $A({\vec{r}}_{0})$, which is buried in an optically diffusive medium illuminated by a temporally varying optical waveform, I(t). The resultant photon absorbed energy, $A({\vec{r}}_{0},t)$ becomes the PA source that produces initial PA waves. An ultrasound transducer whose physical surface is described as $D({\vec{r}}_{d})$ measures those PA waves. For simplicity but without loss of generality, we assume the thermal confinement is fulfilled, which means the PA source, $A({\vec{r}}_{0},t)$ can be separated as spatial and temporal parts, such as $A({\vec{r}}_{0})I(t)$, where $A({\vec{r}}_{0})$ contains an object absorption coefficient, $\mu_{a}$.^1^ From the Green function solution of the PA Helmholtz equation ignoring an object viscosity, the PA spectrum measured by an ultrasound transducer is^1^^,^^23^^,^^24^

$$\overset{˜}{P}(\nu )=[\frac{\Gamma }{4\pi c_{s}^{2}}\int_{\infty }\int_{\infty }D({\overset{\to }{r}}_{d})A({\overset{\to }{r}}_{o})\frac{\exp(-ik|{\overset{\to }{r}}_{d}-{\overset{\to }{r}}_{o}|)}{\left|{\overset{\to }{r}}_{d}-{\overset{\to }{r}}_{o}\right|}d^{3}r_{o}\;d^{3}r_{d}(2\pi i\nu )]\overset{˜}{I}(\nu )\overset{˜}{U}(\nu ),$$ (1)

where $c_{s}$ and Γ are the ultrasound speed and Grüneisen coefficient in the diffusive medium, respectively, which are assumed to be constant. The terms, $\tilde{I}(\nu )$ and $\tilde{U}(\nu )$ indicate spectra of the optical waveform and ultrasound transducer transfer function, respectively. For most PA measurements, a filtering process is applied to measured PA signals, which limits the frequency bandwidth of PA signals to enhance the PA SNR.^12^^–^^14^ Such a linear and shift-invariant filter, q(t), correlated to a PA signal in the time domain, is equivalent to the multiplication of a filter spectrum, $\tilde{Q}(\nu )$ to Eq. (1) in the frequency domain. Defining the square bracket part in Eq. (1) as ${\tilde{O}}_{p}(\nu )$, the finally acquired filtered PA spectrum is simply expressed as

$${\tilde{G}}_{p}(\nu )=\tilde{U}(\nu ){\tilde{O}}_{p}(\nu )\tilde{I}(\nu )\tilde{Q}(\nu ).$$ (2)

Note that the spectrum, ${\tilde{O}}_{p}(\nu )$ is mainly determined by PA imaging systematic parameters, like the physical shape of an ultrasound transducer, $D({\vec{r}}_{d})$ and absorbing energy distribution, $A({\vec{r}}_{0})$.

In reality, a measured PA signal is typically contaminated with a significant amount of noise, such as ultrasound thermal noise. If we denote the noise spectrum by $\tilde{N}(\nu )$, the PA noise spectrum by the linear and shift-invariant filter is described as

$${\tilde{G}}_{n}(\nu )=\tilde{N}(\nu )\tilde{Q}(\nu ).$$ (3)

The PA SNR is defined using Eqs. (2) and (3), which is

$$SNR^{2}=\frac{|\int_{\infty }{\overset{˜}{G}}_{p}(\nu )e^{2\pi i\nu t}d\nu {|}_{\max}^{2}}{\left\langle {\left|\int_{\infty }{\overset{˜}{G}}_{n}(\nu )e^{2\pi i\nu t}d\nu \right|}^{2}\right\rangle }≃\frac{{\left|\int_{\infty }\overset{˜}{G}(\nu )d\nu \right|}^{2}}{\left\langle {\left|\int_{\infty }{\overset{˜}{G}}_{n}(\nu )e^{2\pi i\nu t}d\nu \right|}^{2}\right\rangle },$$ (4)

where the bracket implies temporal averaging and the subscription, *max* means a maximum value of the PA signal. Note that Eq. (4) is for the SNR squared because we derived Eq. (4) from the maximum PA power divided by the PA noise variance. The statistical descriptors, such as mean and variance, should be originally determined by averaging over the ensemble realizations for the maximum PA value. In almost all PA SNR studies, however, the ensemble realizations are unrealistic. The alternative method to calculate those statistical descriptors is to assume the noisy PA data as an ergodic random process,^25^ where the statistical property of the maximum PA values can be represented by other noisy PA values distributed over a temporally extended PA data. We will adopt the ergodicity to all simulation works in this paper. For most PA imaging situations, induced PA waves are measured by an ultrasound transducer after propagating a much longer distance than the PA signal-contributed absorbing object size.^24^^,^^26^ This relatively long propagation distance, L causes a large phase term to ${\tilde{O}}_{p}(\nu )$ in Eq. (2), which makes ${\tilde{O}}_{p}(\nu )$ and thus, ${\overset{˜}{G}}_{p}(\nu )$ rapidly oscillating. For the example of a spherically focused ultrasound transducer, it is reasonable to consider the term, L as the focal length of the transducer. From this concept, we can substitute $\tilde{G}(\nu )\exp(-2\pi i\nu t_{d})$ for ${\tilde{G}}_{p}(\nu )$ in the first equation of Eq. (4), where the time value, $t_{d}$ is $L/c_{s}$. The mathematical description for $\tilde{G}(\nu )$ is similar to Eq. (2) by replacing ${\tilde{O}}_{p}(\nu )$ with $\tilde{O}(\nu )$ that is the bracket part in Eq. (1) excluded the phase term, $\exp(-2\pi i\nu t_{d})$. Then, the maximum PA signal is mainly shown around $t=t_{d}$ since the oscillation rate of the numerator containing the term, $\exp[2\pi i\nu (t-t_{d})]$ is minimal at that time. This well-known stationary phase principle^11^ allows the maximum PA value to be approximated as the integral sum of $\tilde{G}(\nu )$, as shown in the second equation of Eq. (4).

Assuming the PA noise is a stationary random process that is additive to noise-free PA signals, $\tilde{N}(\nu )$ in Eq. (3) is related to the noise power spectral density, $S_{n}(\nu )$ as^25^

$$\langle \overset{˜}{N}(\nu ){\overset{˜}{N}}^{*}(\nu^{\prime })\rangle =S_{n}(\nu )\delta (\nu -\nu^{\prime }),$$ (5)

where $\delta (\nu -\nu^{\prime })$ is a Dirac delta function. For a white Gaussian thermal noise that is reasonably acceptable in most PA measurement situations,^7^^,^^8^^,^^13^^,^^14^ the power spectral density in Eq. (5) can be assumed to be constant over the frequency band. However, we will continue with the frequency-dependent power spectral density form in Eq. (5) for generality. Substituting Eqs. (2) and (3) to Eq. (4) and processing the noise variance term in the denominator of Eq. (4) with Eq. (5), the SNR squared in Eq. (4) is approximated to

$${\mathrm{SNR}}^{2}=\frac{{\left|\int_{\infty }\overset{˜}{U}(\nu )\overset{˜}{O}(\nu )\overset{˜}{I}(\nu )\overset{˜}{Q}(\nu )d\nu \right|}^{2}}{\int_{\infty }S_{n}(\nu ){\left|\overset{˜}{Q}(\nu )\right|}^{2}d\nu }.$$ (6)

Note that the statistical averaging terms in Eq. (4) disappear in the SNR expression in Eq. (6). It was already shown in the previous literature^23^ that the SNRs in Eq. (6) approach to the SNRs of the first expression in Eq. (6) as the number of simulated noisy PA data is increased.

## 3 SNR Ratio for Single Pulse and Chirp Waveforms

The SNR of Eq. (6) is valid for arbitrary waveform and filtering spectra, $\tilde{I}(\nu )$ and $\tilde{Q}(\nu )$. For the PA radar, the chirp waveform of

$$i_{c}(t)=\frac{E_{c}}{T}[1+\cos(2\pi \nu_{0}t+\pi \beta t^{2})]\Pi (\frac{t}{T}),$$ (7)

is usually considered,^11^^,^^12^ where T, $\nu_{0}$, and β indicate the duration, center frequency, and frequency sweep rate of the chirp waveform, respectively. The terms $E_{c}$ and Π(t) are the chirp waveform fluence and temporal rectangular function. The bandwidth of the chirp waveform in Eq. (7) is βT, which is started at $\nu_{1}=\nu_{0}-\beta T/2$ and ended at $\nu_{2}=\nu_{0}+\beta T/2$. For the PA excitation from a pulse waveform, we assume the Gaussian-shaped pulse, $i_{p}(t)$ with the pulse width, τ and pulse fluence, $E_{p}$, which is

$$i_{p}(t)=\frac{E_{p}}{\tau \sqrt{\pi }}\;\exp(-\frac{t^{2}}{\tau^{2}}).$$ (8)

The maximum fluence of incident optical waveforms to the human skin is limited by the ANSI MPE, which is

$$\left\{ \begin{array}{l} 0.02C_{A}\\ 1.1C_{A}T^{1/4} \end{array}\;\text{for }\{\begin{array}{l} 10^{-9}\leq T\leq 10^{-7}\\ 10^{-7}\leq T\leq 10 \end{array}\;s,\right.$$ (9)

respectively.^6^ The unit for the ANSI MPE in Eq. (9) is $J\cdot {\mathrm{cm}}^{-2}$. Note that we express the waveform duration as the notation, T for the ANSI MPE although we used the two different notations, T and τ for chirp and pulse waveform durations in Eqs. (7) and (8) to differentiate them. The constant value, $C_{A}$ is wavelength-dependent, which is 1 for 400 to 700 nm and $10^{0.002(\lambda -700)}$ for 700 to 1050 nm. For simplicity without loss of generality, we approximate this value as 1 considering visible light PA excitations.

For the chirp waveform, the filter spectrum, $\tilde{Q}(\nu )$ is determined from the concept of cross correlation of the original chirp waveform of Eq. (7), which is $\tilde{Q}(\nu )={\tilde{I}}^{*}(\nu )$, where the symbol, * means complex conjugate. Resulting from this matched filtering process, the filtered waveform spectrum, $\overset{˜}{I}(\nu )\overset{˜}{Q}(\nu )$ can be approximated to a rectangular bandpass filter of the bandwidth, βT if the time-bandwidth product, $\beta T^{2}$ is more than 30.^12^ The $\beta T^{2}$ value for typical chirp waveforms considered in PA radar is at least a few hundred. For the pulse waveform, the filter spectrum, $\tilde{Q}(\nu )$ is chosen as a rectangular bandpass filter for simplicity. We assume the bandwidth of this bandpass filter is the same as that of the chirp spectrum for the study of SNR comparison. Since the magnitude of $\tilde{Q}(\nu )$ does not affect the SNR, as easily known in in Eq. (6), we choose the unity-magnitude filter spectra to make noise variances of both waveform cases coincident. The analytic form of the unity-magnitude matched filter spectrum for the chirp waveform of Eq. (7) was derived in the previous literature.^12^ Substituting waveforms and filter spectra explained by now to Eq. (6), we can derive the SNR forms of pulse and matched filtered PA signals for a given $\tilde{U}(\nu )\tilde{O}(\nu )$, which are denoted as ${\mathrm{SNR}}_{p}$ and ${\mathrm{SNR}}_{c}$, respectively. Then, the ratio of these SNRs becomes

$$\frac{{\mathrm{SNR}}_{p}}{{\mathrm{SNR}}_{c}}=\frac{2{\mathrm{TE}}_{p}}{E_{c}}\sqrt{\beta }\gamma (\tau ,\Delta \nu ),$$ (10)

where

γ(τ,Δν)≡|∫ν1ν2 Re[U˜(ν)O˜(ν)]exp(−π2τ2ν2)dν||∫ν1ν2 Re[U˜(ν)O˜(ν)]dν|. (11)

In Eq. (11), the term, Re[U˜(ν)O˜(ν)] means the real part of $\tilde{U}(\nu )\tilde{O}(\nu )$. Since a measured PA signal is real, $\tilde{U}(\nu )\tilde{O}(\nu )$ is Hermitian, which means $\tilde{U}(\nu )\tilde{O}(\nu )={\tilde{U}}^{*}(-\nu ){\tilde{O}}^{*}(-\nu )$. Originally, each of the numerator and denominator in γ(τ,Δν) in Eq. (11) contains the integral for the negative frequency range of $-\nu_{2}$ to $-\nu_{1}$. Applying the Hermitian characteristic simplifies the γ(τ,Δν), as expressed in Eq. (11). Also, the DC frequency value of the $\tilde{U}(\nu )\tilde{O}(\nu )$ is usually zero due to the spatial filtering and characteristics of an ultrasound transducer.^13^^,^^24^^,^^27^ However, the power spectral density of the ultrasound thermal noise is flat including a DC component. This implies that the DC component in the filtered waveform spectrum, $\tilde{I}(\nu )\tilde{Q}(\nu )$ contributes only to the denominator of Eq. (6), which always reduces the SNR. For the pulse waveform, the lowest frequency of bandpass filters is typically far from a DC, thus this DC-induced SNR reduction does not occur. For the chirp waveform, however, the strong DC term exists in $\tilde{Q}(\nu )$ if $\tilde{Q}(\nu )={\tilde{I}}^{*}(\nu )$, which significantly reduces the ${\mathrm{SNR}}_{c}$.^23^ To avoid the DC-induced SNR reduction in PA radar, we dealt with the matched filter spectrum whose DC term is intentionally removed, which was already considered in the derivation of Eqs. (10) and (11).

**Fig. 1:**
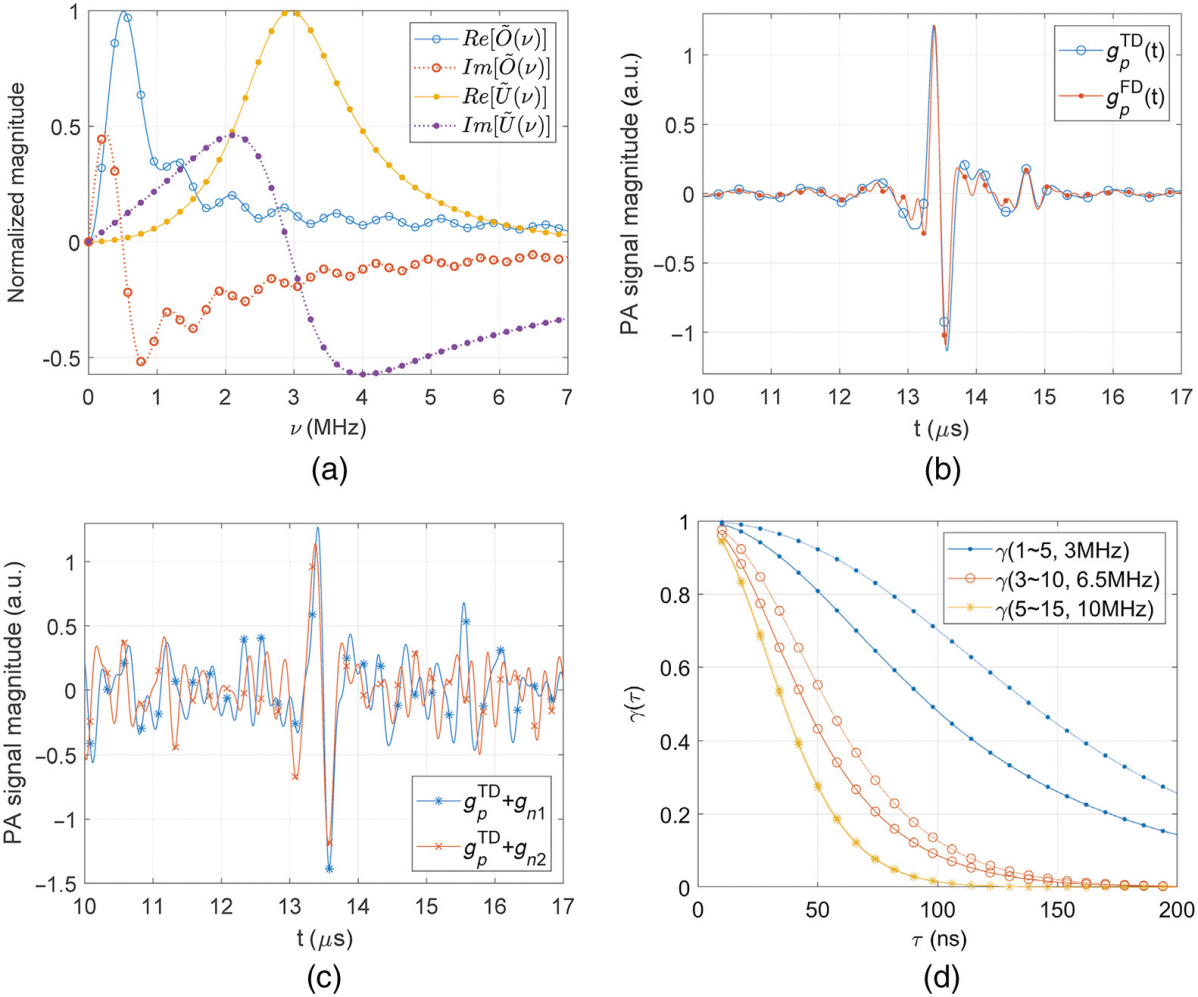
(a) Simulated real and imaginary parts of $\tilde{O}(\nu )$ and $\tilde{U}(\nu )$, e.g., PA macroscopic imaging configuration with spherically focused ultrasound transducer and sphere absorbing object. Simulated (b) mean and (c) thermal noisy PA signals from 100-ns pulse and matched filtering with 1-ms chirp waveforms. (d) The variation of $\gamma (\Delta \nu ,\nu_{c})$ to a pulse width (τ) for different bandwidths (Δν) and central frequencies ($\nu_{c}$) of $\tilde{U}(\nu )$ and $\tilde{O}(\nu )$ functions.

As the example examining the SNR ratio of Eq. (10) affected by the term, γ(τ,Δν) of Eq. (11), we set the specific PA macroscopic imaging configuration, where the spherically focused ultrasound transducer of 0.35 numerical aperture focuses on sphere-shaped absorbing objects buried in a diffusive medium.^24^^,^^26^ Assuming an exponentially decayed irradiance in the absorbing object of a $10\;{\mathrm{cm}}^{-1}$ absorption coefficient, the PA spectrum, ${\tilde{O}}_{p}(\nu )$, the bracket part in Eq. (1), can be acquired by directly simulating the PA Helmholtz equation.^24^ We also simulate the ultrasound transducer transfer function, $\tilde{U}(\nu )$ from the simplified equation similar to the Krimholtz–Leedom–Matthaei model.^13^^,^^23^^,^^24^ For the 2-mm diameter sphere absorbing object, simulated real and imaginary parts of $\tilde{O}(\nu )$ and $\tilde{U}(\nu )$ normalized by each maximum magnitude, are shown in Fig. 1(a). Here, the phase term, $\exp(-2\pi i\nu t_{d})$, where $t_{d}=20\;\mathrm{mm}$, are intentionally excluded from ${\tilde{O}}_{p}(\nu )$ for the clear demonstration and the central frequency, $\nu_{c}$ is set to 3 MHz for $\tilde{U}(\nu )$. Considering $\tilde{O}(\nu )\tilde{U}(\nu )$ with the bandwidth of 1 to 5 MHz in Fig. 1(a), PA signals from the 100-ns pulse and 1-ms chirp waveforms are simulated by inverse Fourier transforming Eq. (2), which are shown in Fig. 1(b). The maximum magnitude of the matched filtered PA signal is scaled to fit to that of the pulse PA signal for comparison. Those PA signals in time- and frequency-domains are slightly different because the spectral bandpass function resulting from the matched filtering is not exactly the same as the rectangular one. Notice that there are the first and second peaks at t∼13.39 and 14.73  μs for both signals, which are caused by initially induced PA waves from the top and bottom surfaces of the 2-mm diameter sphere absorbing object, respectively. As shown in Fig. 3(c), thermal noise-contaminated PA signals can also be simulated by adding noise generated from Eq. (3), where the noise power spectral density is assumed to be constant.

If the pulse width, τ is very short like a few nanoseconds or less, the exponential function in the numerator of γ(τ,Δν) in Eq. (11) becomes nearly flat in the bandwidth, which indicates γ(τ,Δν)≈1. Under this condition, the SNR ratio of Eq. (10) becomes equivalent to Petschke and La Riviere result.^12^ If the pulse duration is much longer than a few nanoseconds, which is typical in pulse PA excitations using LEDs or LDs, the γ(τ,Δν) becomes smaller than 1. We plot $\gamma (\Delta \nu ,\nu_{c})$ of Eq. (11) in Fig. 1(d) for different bandwidth ranges ($\Delta \nu =\nu_{2}-\nu_{1}$) and central frequencies ($\nu_{c}$), where $\tilde{O}(\nu )$ and $\tilde{U}(\nu )$ in Fig. 1(a) for those different Δν and $\nu_{c}$ values were considered. For comparison, we co-plot the correct γ functions that can be derived by dividing the exactly calculated SNR ratios with the term, $2{\mathrm{TE}}_{p}\sqrt{\beta }/E_{c}$ in Eq. (10). The exact SNR ratios were directly calculated from simulated PA noisy signals, as exampled in Figs. 1(b) and 1(c). Figure 1(d) shows that there is some difference between γ functions of the correct and Eq. (11), which are denoted as solid and dotted lines, but the amount of the difference is reduced as the central frequency and bandwidth increase. Overall, it is seen in Fig. 1(b) that the γ functions are significantly reduced as the pulse width, τ increases, as easily understood in Eq. (11). Also, the rate of the γ reduction to τ is more accelerated for a higher central frequency of $\tilde{U}(\nu )$.

**Fig. 2:**
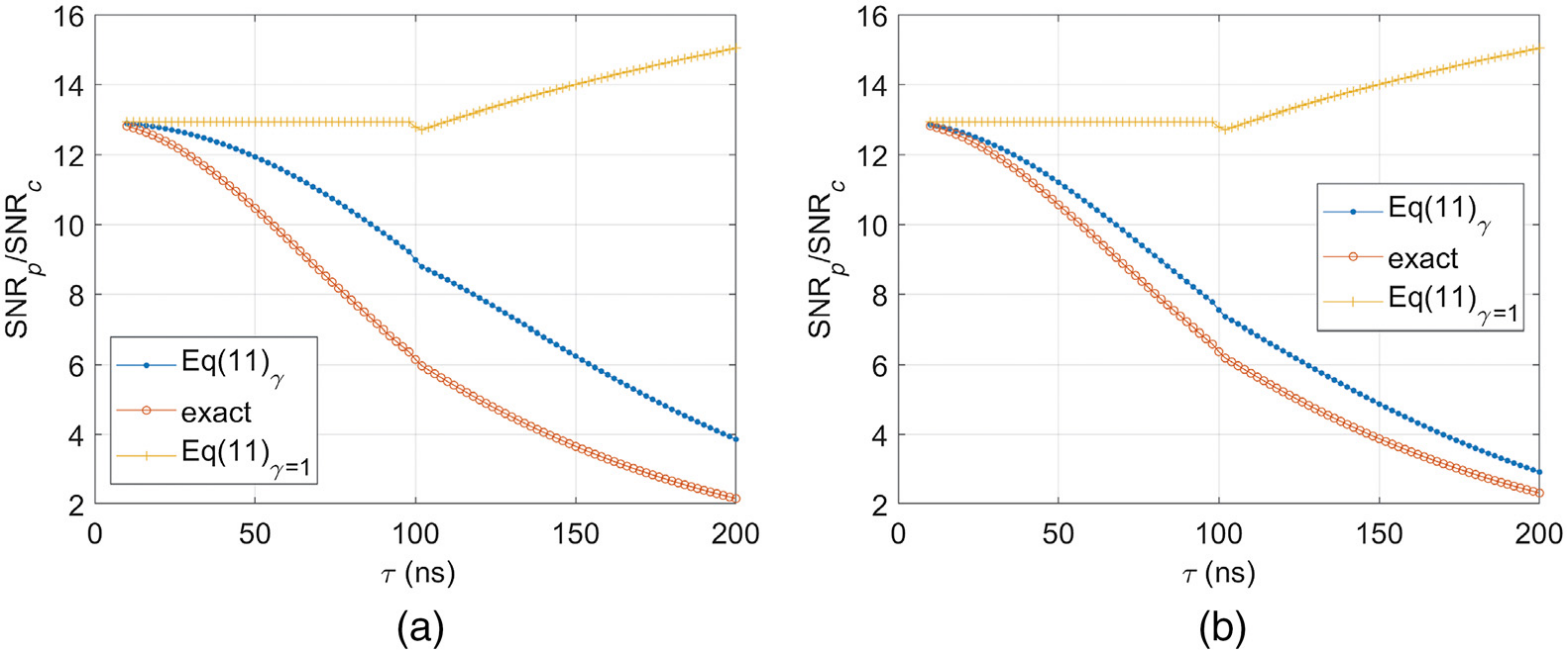
SNR ratios calculated from Eq. (11) with and without γ in Eq. (12) to a pulse width (τ) for the PA macroscopic imaging to sphere absorbing objects of dimeters of (a) 2 and (b) 4 mm. The term, exact in the legend indicates SNR ratios from exactly simulated γ functions.

Figures 2 shows SNR ratios of Eq. (10) for single pulse and matched filtered PA signals assuming the waveform fluence equals to the ANSI MPE of Eq. (9). The all SNR ratios from the γ(τ,Δν) of Eq. (11), exactly simulated γ, and γ=1 are shown for verification and comparison. We set the chirp waveform duration as 1 ms and change the pulse width from 10 to 200 ns, as shown in Fig. 2. At the pulse width of 100 ns, we considered the average pulse waveform fluence from Eq. (9). In both Figs. 2(a) and 2(b), which are for sphere absorbing objects of 2- and 4-mm diameters, respectively, it can be seen that the SNR ratios from γ=1, which have been regarded to be correct in the previous literature,^12^ significantly deviate from the exact ones as the pulse width increases. The SNR ratios with consideration of γ(τ,Δν) are quickly decreased as the pulse width increases in both Figs. 2(a) and 2(b). There is some difference between SNR ratios from the exact and Eq. (10), which is mainly caused by the γ difference, as shown in Fig. 1(d). Although not shown here, we observed that the difference between those two SNR ratios becomes gradually smaller as the diameter of the absorbing object increases more, as notified in Fig. 2. This implies that the stationary phase approximation that was assumed for the derivation of Eqs. (6), (10), and (11) does not work well for a point-like absorbing object located near the focal point of a focused ultrasound transducer. For such a PA imaging configuration, the range of main frequency components composing PA signals is relatively narrow so that the fast oscillating parts in $\exp[2\pi i\nu (t-t_{d})]$ as t is away from $t_{d}$ cannot be effectively averaged out by the integration in the frequency range. However, Fig. 2 indicates that the theoretical results of Eqs. (10) and (11) are highly acceptable estimations of the actual SNR ratios compared to the conventional ones, which also provide intuitive understanding. For instance, Eqs. (10) and (11) clearly show the low-pass filtering to $\tilde{O}(\nu )\tilde{U}(\nu )$ by the pulse PA excitation. This low-pass filtering is intensified as a pulse duration increases, which accelerates the reduction of the SNR ratio.

## 4 SNR Ratio for Pulse Train and Chirp Waveforms

### 4.1 Pulse Fluence-Dependent SNR Ratio

We suppose a pulse train waveform, where there are N number of identical pulses of the width, τ with a fixed duty cycle in the duration, T. Assuming $10^{-7}\leq T\leq 10\;s$, the fluence conditions for the pulse train based on the ANSI MPE in Eq. (9) are

$$\left\{ \begin{array}{l} 0.02C_{p}N\leq 1.1T^{1/4}\\ 1.1C_{p}\tau^{1/4}N\leq 1.1T^{1/4} \end{array}\;\text{for }\{\begin{array}{l} 10^{-9}\leq \tau \leq 10^{-7}\\ 10^{-7}\leq \tau \leq 10 \end{array}\;s,\right.$$ (12)

where the pulse fluence reduction rate, $C_{p}(\leq 1)$ is intentionally inserted to represent that the fluence per pulse is smaller than the ANSI MPE for a single pulse in Eq. (9). As typical in most LED/LD-based pulse train PA systems, we assume the $C_{p}$ value is the same for all individual pulses in the pulse train. From Eq. (12), the pulse train fluence reduction factor, $C_{pt}$ can be defined as

$$C_{\mathrm{pt}}=\{\begin{array}{l} 0.02C_{p}N/(1.1T^{1/4})\\ 1.1C_{p}\tau^{1/4}N/(1.1T^{1/4}) \end{array}\;\text{for }\{\begin{array}{l} 10^{-9}\leq \tau \leq 10^{-7}\\ 10^{-7}\leq \tau \leq 10 \end{array}\;s,$$ (13)

i.e., the $C_{p}$ and $C_{\mathrm{pt}}$ indicate fluence reduction factors for a single pulse and pulse train, respectively. It is noteworthy that the factor, $C_{\mathrm{pt}}$ in Eq. (13) is the function of pulse train parameters, such as τ and T, although $C_{p}$ is constant. Also, even though $C_{p}$ is much smaller than 1, $C_{\mathrm{pt}}$ could be 1, meaning the pulse train duration is extended to reach the ANSI MPE. The fluence condition in Eq. (12) derives the condition for the pulse number of the pulse train, which is

$$N\leq N_{\max}=\{\begin{array}{l} 1.1T^{1/4}/(0.02C_{p})\\ T^{1/4}/(C_{p}\tau^{1/4}) \end{array}\;\text{for }\{\begin{array}{l} 10^{-9}\leq \tau \leq 10^{-7}\\ 10^{-7}\leq \tau \leq 10 \end{array}\;s.$$ (14)

Since at least one pulse should exist, there are the inherent conditions behind Eq. (14) from $N_{\max}\geq 1$, which are $1.1T^{1/4}\geq 0.02C_{p}$ and $T^{1/4}\geq C_{p}\tau^{1/4}$, respectively. Since the PA SNR excited by the pulse train, ${\mathrm{SNR}}_{pt}$ is increased by averaging pulse PA signals, it is reasonable to consider ${\mathrm{SNR}}_{\mathrm{pt}}={\mathrm{SNR}}_{p}\sqrt{N}$, where ${\mathrm{SNR}}_{p}$ is the SNR of a single pulse PA signal. Assuming both pulse train and chirp waveform fluences are maximized to the ANSI MPE, $1.1T^{1/4}$ (i.e., $C_{pt}=1$ or, equivalently, $N=N_{\max}$), the SNR ratio of pulse train and matched filtered PA signals by chirp waveforms can be derived from Eqs. (10) and (14), which is

$$\frac{{\mathrm{SNR}}_{\mathrm{pt}}}{{\mathrm{SNR}}_{c}}=\{\begin{array}{l} 2\sqrt{0.02/1.1}\sqrt{C_{p}\Delta \nu }T^{3/8}\gamma (\tau ,\Delta \nu )\\ 2\tau^{1/8}\sqrt{C_{p}\Delta \nu }T^{3/8}\gamma (\tau ,\Delta \nu ) \end{array}\;\text{for }\{\begin{array}{l} 10^{-9}\leq \tau \leq 10^{-7}\\ 10^{-7}\leq \tau \leq 10 \end{array}\;s.$$ (15)

Here, the first SNR ratio expression is the same as the second one if the term, $\sqrt{0.02/1.1}$ is substituted by $\tau^{1/8}$. However, when τ=100  ns, $\tau^{1/8}∼0.1334$, which is slightly different from $\sqrt{0.02/1.1}∼0.1348$. Except the $\sqrt{C_{p}}$ and γ(τ,Δν) terms, the first expression in Eq. (15) is the same as the previously reported result that had suggested ${\mathrm{SNR}}_{\mathrm{pt}}$ was 20 to 30 dB higher than ${\mathrm{SNR}}_{c}$.^12^ It is noteworthy that the additional terms, $\sqrt{C_{p}}$ and γ(τ,Δν) could significantly reduce the SNR ratio for LED/LD-based PA systems, as implied in Eq. (11) and Figs. 1 and 2.

The maximum pulse number in Eq. (14) for the derivation of Eq. (15) implies the repetition rate of pulse train waveforms could be impractically high. To circumvent this impractical assumption, the fixed repetition rate of a pulse train, R=N/T, can be introduced, which derives the practically applicable SNR ratio as

$$\frac{{\mathrm{SNR}}_{\mathrm{pt}}}{{\mathrm{SNR}}_{c}}=\{\begin{array}{l} 2(0.02C_{p}/1.1)\sqrt{R\Delta \nu }T^{3/4}\gamma (\tau ,\Delta \nu )\\ 2C_{p}\tau^{1/4}\sqrt{R\Delta \nu }T^{3/4}\gamma (\tau ,\Delta \nu ) \end{array}\;\text{for }\{\begin{array}{l} 10^{-9}\leq \tau \leq 10^{-7}\\ 10^{-7}\leq \tau \leq 10 \end{array}\;s.$$ (16)

Considering the ANSI MPE condition in Eq. (12) and $N_{\max}$ in Eq. (14), the pulse repetition rate in Eq. (16) has the limit condition of

$$R\leq R_{\max}=\{\begin{array}{l} 1.1/(0.02C_{p}T^{3/4})\\ 1/(C_{p}\tau^{1/4}T^{3/4}) \end{array}\;\text{for }\{\begin{array}{l} 10^{-9}\leq \tau \leq 10^{-7}\\ 10^{-7}\leq \tau \leq 10 \end{array}\;s.$$ (17)

For both Eqs. (16) and (17), the terms, 0.02/1.1 and $\tau^{1/4}$ are the only different parts between the first and second expressions for $10^{-9}\leq \tau \leq 10^{-7}$ and $10^{-9}\leq \tau \leq 10^{-7}\;s$. It is noteworthy that achieving $R=R_{\max}$ is equivalent to $N=N_{\max}$ in a given pulse train duration, which makes Eq. (16) the same as Eq. (15). A typical repetition rate in LED/LD-based pulse train PA systems is below a few KHz, which are much smaller than $R_{\max}$ of Eq. (17).^15^^–^^20^ For the specialized LED/LD module having a very high repetition rate, like a few hundred KHz, the $C_{p}$ becomes very low, like a few ten-thousandth,^21^ which makes $R_{\max}$ increased up to a few tens of MHz. Therefore, it is reasonable to consider that achieving the maximum repetition rate or, equivalently, the maximum pulse number is almost impossible for almost all practical LED/LD-based PA systems. Equation (16) indicates that if the pulse repetition rate is fixed and smaller than $R_{\max}$ in Eq. (17), the SNR ratio is proportional to $C_{p}$. This implies the possibility that the SNR ratio could be further reduced in LED/LD-based low optical fluence pulse PA excitations.

**Fig. 3:**
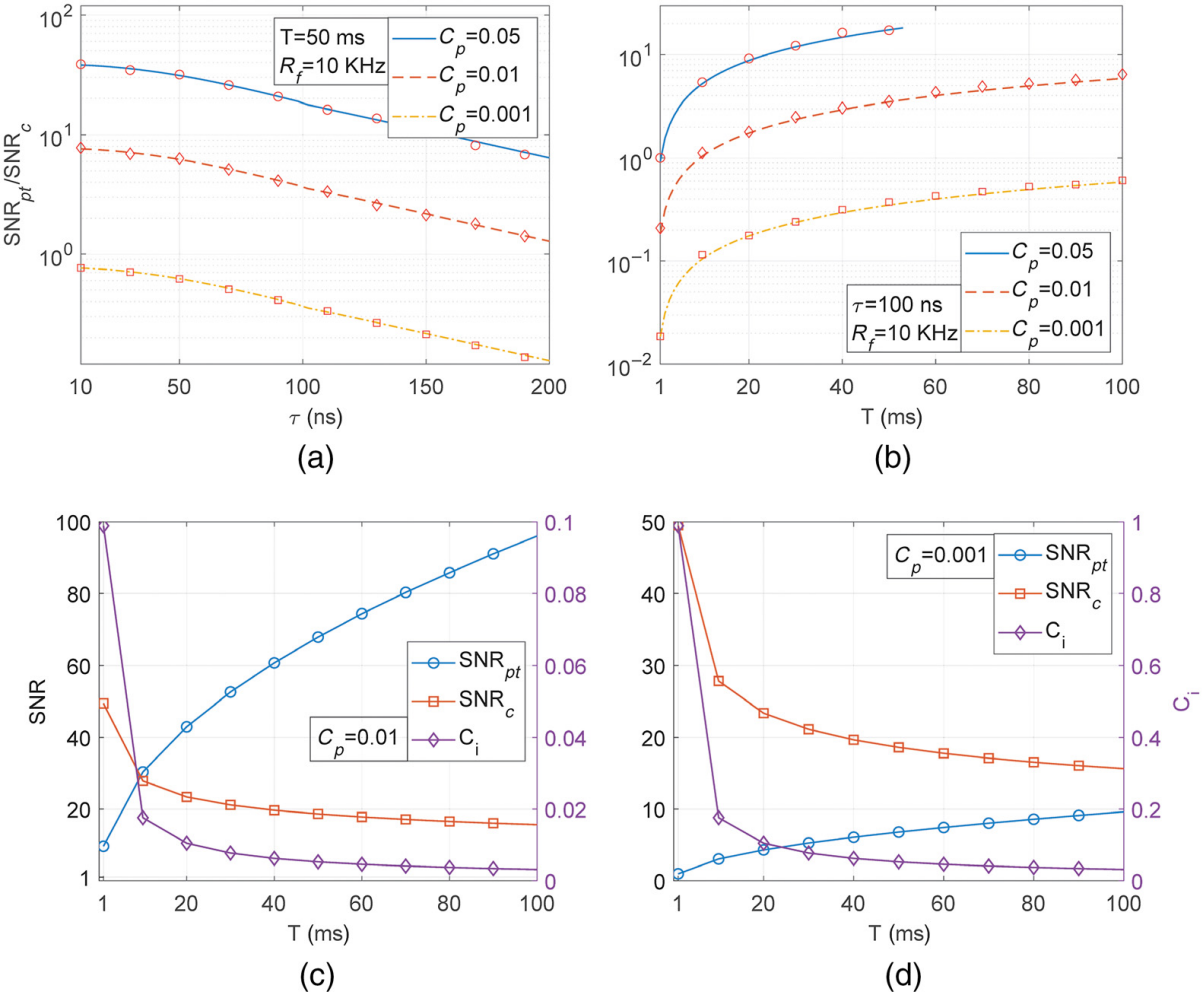
For different values of $C_{p}$, the SNR ratios of pulse train and matched filtered PA signals with chirp waveforms for fixed (a) waveform duration of 50 ms and (b) pulse width of 100 ns when the pulse repetition rate is fixed as 10 KHz. The y axes of (a) and (b) are shown as log-scaled for clarity. The simulated SNRs of pulse train and matched filtered PA signals are separately shown for $C_{p}$ = (c) 0.01 and (d) 0.001.

Figures 3(a) and 3(b) show the log-scaled SNR ratios calculated from Eq. (16) for the same PA macroscopic imaging configuration considered in Figs. 1 and 2 when the pulse train repetition rate is fixed to 10 KHz. The $C_{p}$ values are differently chosen as 0.05, 0.01, and 0.001 in Fig. 3 considering actual LED/LD-based pulse train PA systems in the previous literature. Brute-force simulated SNR ratios are co-plotted for verification, which are indicated as open circle, diamond, and rectangular dots. For the brute-force simulation, the noisy PA signals from pulse and chirp waveforms are simulated, as exemplified in Figs. 1(b) and 1(c), where the maximum mean signal and noise standard deviation values are calculated to extract SNRs. For pulse train waveforms, especially, we summed simulated noisy pulse PA signals N times, where N is the natural number determined by the minimum between R×T and $N_{\max}$ in Eq. (14). The SNR ratios in Fig. 3(a) are dramatically decreased as the pulse width of pulse trains is increased, which is caused by the characteristics of γ(τ,Δν). Also, Fig. 3(b) indicates the trend that SNR ratios proportionally increase with the factor of $T^{3/4}$, as derived in Eq. (16). In Fig. 3(b), the $C_{p}=0.05$ case is shown only up to T∼53  ms because the pulse train fluence factor, $C_{pt}$ exceeds 1 for T>53  ms. Figures 3(a) and 3(b) clearly demonstrate that the SNRs of matched filtered PA signals could be comparable to and even higher than those of pulse train PA signals for low optical power PA systems. For better understanding, the variation of brute-force simulated SNRs of the pulse train (${\mathrm{SNR}}_{\mathrm{pt}}$) and matched filtered PA signals (${\mathrm{SNR}}_{c}$) to T are separately plotted in Figs. 3(c) and 3(d) for $C_{p}=0.01$ and 0.001. It is noteworthy that the SNRs of matched filtered PA signals are almost the same in both Figs. 3(c) and 3(d), but the SNRs of pulse train PA signals are significantly reduced as the $C_{p}$ decreases. The reason for the unchanged SNRs of matched filtered PA signals is that the chirp waveform irradiance is high enough to reach the ANSI MPE for the 1 to 100 ms waveform duration range even $C_{p}$ is reduced to 0.001. The chirp waveform irradiance condition related to pulse trains will be described in the next section with the definition of the $C_{i}$ value shown in Figs. 3(c) and 3(d).

### 4.2 Lower and Upper Bounds for SNR Ratios

Since the same optical component (i.e., LED or LD) radiates either pulse train or chirp waveforms, the irradiance of a chirp waveform must be determined based on the condition of the single pulse energy of a pulse train. From this concept, it is reasonable to consider that the maximum chirp waveform irradiance is equivalent to the pulse power divided by the pulse width, τ, which is

$$\frac{E_{c}}{T}=\{\begin{array}{l} 0.02C_{p}/\tau \\ 1.1C_{p}\tau^{1/4}/\tau \end{array}\;\text{for }\{\begin{array}{l} 10^{-9}\leq \tau \leq 10^{-7}\\ 10^{-7}\leq \tau \leq 10 \end{array}\;s.$$ (18)

Note that these chirp waveform irradiances are proportional to the pulse fluence reduction factor, $C_{p}$. For almost all LED/LD-based PA systems, the duration of pulse train and chirp waveforms is much longer than 100 ns. Thus, applying the ANSI MPE irradiance condition, $1.1T^{1/4}/T=1.1T^{-3/4}$ to Eq. (18) derives the limiting condition for T, which is

$$T_{\max}^{c}=\{\begin{array}{l} {\left(\frac{1.1\tau }{0.02C_{p}}\right)}^{4/3}\\ \tau /C_{p}^{4/3} \end{array}\text{for }\{\begin{array}{l} 10^{-9}\leq \tau \leq 10^{-7}\\ 10^{-7}\leq \tau \leq 10 \end{array}\;s.$$ (19)

**Fig. 4:**
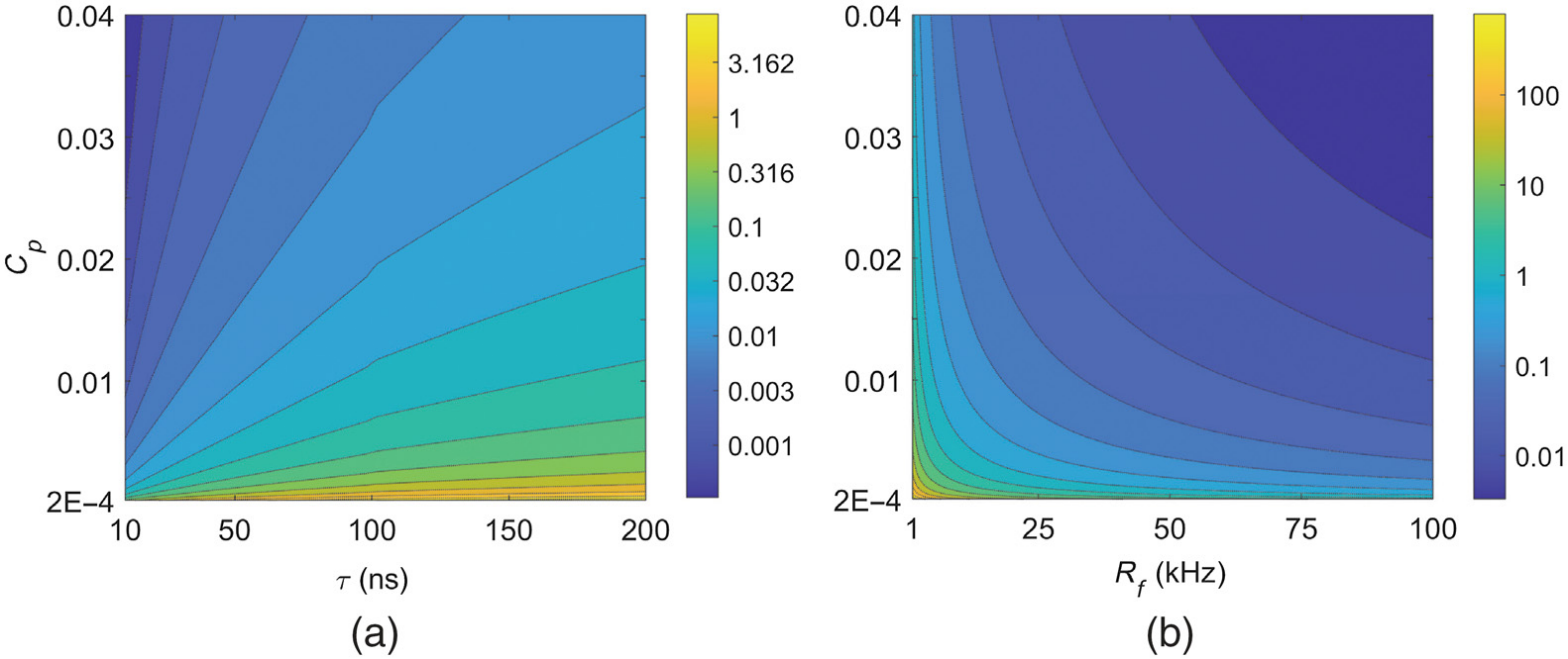
The distribution of waveform durations of (a) $T_{\max}^{c}$ and (b) $T_{\max}^{p}$ for the fluence reduction factor, $C_{p}$; pulse width, τ; and maximum repetition rate, $R_{f}$. The units for colorbars of (a) and (b) are milliseconds and seconds, respectively.

The $T_{\max}^{c}$ must be longer than the individual pulse duration, τ of the pulse train. The second expression in Eq. (19) always satisfies this requirement by $C_{p}<1$. For the first expression in Eq. (19), the requirement leads to the additional restriction, ${\left(1.1/0.02\right)}^{4}\geq C_{p}^{4}\tau^{-1}$, which is equivalent to the condition for $N_{\max}\geq 1$ in Eq. (14). This additional restriction is fulfilled for almost all LED/LD-excited PA measurements, where $C_{p}$ is usually smaller than 0.1. For example, for τ=10 and 20 ns, the upper bounds for $C_{p}$ to fulfill the additional restriction are 0.549 and 0.654, respectively. Figure 4(a) shows the contoured $T_{\max}^{c}$ distribution calculated from Eq. (19) for $0.0002\leq C_{p}\leq 0.04$ and 10≤τ≤200  ns. For the pulse width, τ=100  ns, the $T_{\max}^{c}$ values are the averaged ones from two expressions in Eq. (19). As shown later in this paper, these ranges for $C_{p}$ and τ values were determined considering the real LED/LD-based pulse train PA systems in the previous literature. It is shown in Fig. 4(a) that the $T_{\max}^{c}$ gets longer as $C_{p}$ decreases and τ increases. It is also observed that $T_{\max}^{c}$ values are increased up to milliseconds for those very low $C_{p}$ values.

Since the chirp waveform irradiance is limited as Eq. (18) and the pulse train SNR is decreased as T gets smaller, there is no practical benefit to further reduce the waveform duration from $T_{\max}^{c}$ for both pulse train and chirp waveforms. Assuming $T=T_{\max}^{c}$, the SNR ratio in Eq. (16) becomes

$$\frac{{\mathrm{SNR}}_{pt}}{{\mathrm{SNR}}_{c}}=2\tau \sqrt{R\Delta \nu }\gamma (\tau ,\Delta \nu ).$$ (20)

Although the SNR ratio and $T_{\max}^{c}$ expressions are different for different τ ranges in Eqs. (16) and (19), the SNR ratios separately derived for each τ range are coincident each other, which is Eq. (20). Equation (20) is the lower bound of the SNR ratio of Eq. (16), which is regardless of $C_{p}$. For most LED/LD-based PA measurements, the SNR ratio in Eq. (20) is much lower than 1, which implies the matched filtering with chirp waveforms achieves much better SNRs than pulse train waveforms as long as $T=T_{\max}^{c}$. For example, τ=70  ns, R=4  KHz, and Δν=8.1  MHz,^19^ the SNR ratio part without γ(τ,Δν) in Eq. (20) is just 0.025. Considering the typical γ(τ,Δν) value is much smaller than 1, as shown in Eq. (11) and Fig. 1(d), it is easily expected the SNR ratio in Eq. (20) is significantly lower than 1 for that PA system.

It is possible to extend the pulse train duration to average more pulse PA signals to increase the SNR. The chirp waveform duration longer than $T_{\max}^{c}$ is conceptually equivalent to further decreasing the chirp waveform irradiance from Eq. (18) to fulfill the ANSI MPE condition. The irradiance reduction factor, $C_{i}\leq 1$ can be introduced, which is multiplied to the chirp waveform irradiance of Eq. (18) to indicate how much the irradiance is reduced for $T\geq T_{\max}^{c}$. If the similar procedure of deriving Eq. (19) is performed with the consideration of $C_{i}$ to Eq. (18), the condition of

$$C_{i}(T)={\left(T_{\max}^{c}/T\right)}^{3/4}$$ (21)

can be extracted. The proportionality of the SNR ratio to $T^{3/4}$ in Eq. (16) indicates that the condition, $T\geq T_{\max}^{c}$ increases the SNR ratio of Eq. (20) as the factor of $1/C_{i}$. For example, if T is ten times longer than $T_{\max}^{c}$, the irradiance reduction factor, $C_{i}$ is ${\left(1/10\right)}^{3/4}=0.1778$, which means the SNR ratio of Eq. (20) increases 1/0.1778≃5.625 times. All $C_{i}$ values in Figs. 3(c) and 3(d) are lower than 1, which indicates all chirp waveforms considered in Fig. 3 satisfy the ANSI MPE. Also, $C_{i}∼1$ for τ=100  ns, $C_{p}=0.001$, and T=1  ms in Fig. 3(d) implies $T_{\max}^{c}∼1\;\mathrm{ms}$ for this condition. The $C_{i}$ values in Fig. 3(a) are much smaller than 1, which means the chirp waveform irradiance can be increased for waveform durations shorter than 1 ms, where the SNR ratio is further decreased. However, there are practical limitations to shortening the chirp waveform duration, which might be on the order of tens of microseconds in a typical PA radar.^13^^,^^27^

If the maximum pulse repetition rate in a LED/LD-based pulse PA system is noted as $R_{f}$, the maximum pulse train duration can be calculated from Eq. (17), which is

$$T\leq T_{\max}^{p}=\{\begin{array}{l} {\left(\frac{1.1}{0.02C_{p}R_{f}}\right)}^{4/3}\\ {\left(C_{p}R_{f}\right)}^{-4/3}\tau^{-1/3} \end{array}\;\text{for }\{\begin{array}{l} 10^{-9}\leq \tau \leq 10^{-7}\\ 10^{-7}\leq \tau \leq 10 \end{array}\;s.$$ (22)

The waveform duration, $T_{\max}^{c}$ in Eq. (19) was extracted in terms of a chirp waveform under the ANSI MPE based on the chirp waveform irradiance of Eq. (18). The waveform duration, $T_{\max}^{p}$ in Eq. (22) was extracted in terms of a pulse train waveform under the ANSI MPE based on the fixed pulse repetition rate. Figure 4(b) shows contoured $T_{\max}^{p}$ distribution for the pulse width range of $10^{-9}\leq \tau \leq 10^{-7}\;s$ when the ranges of $R_{f}$ and $C_{p}$ are $1\leq R_{f}\leq 100\;\mathrm{KHz}$ and $0.0002\leq C_{p}\leq 0.04$. Although Fig. 4(b) shows the $T_{\max}^{p}$ can be decreased up to ∼0.01  s for $C_{p}∼0.04$ and $R_{f}∼100\;\mathrm{KHz}$, such a small value for $T_{\max}^{p}$ is impractical because a relatively high-power LED/LD module achieving $C_{p}\geq 0.04$ typically has a low repetition rate below a few KHz ^18^^,^^22^

Assuming $T=T_{\max}^{p}$ for both pulse train and chirp waveforms, substituting $T_{\max}^{p}$ of Eq. (22) to T in Eq. (16) produces the SNR ratio expression, which is

$$\frac{{\mathrm{SNR}}_{pt}}{{\mathrm{SNR}}_{c}}=2\sqrt{\Delta \nu /R_{f}}\gamma (\tau ,\Delta \nu ).$$ (23)

Similar to the derivation procedure for Eq. (20), the SNR ratios separately derived for each τ range are coincident each other, which is Eq. (23). The SNR ratio in Eq. (23) is independent of $C_{p}$ and usually higher than 1 for a typical LED/LD-based PA imaging configuration unless the γ(τ,Δν) value is very small. However, it would be inappropriate to intentionally reduce the chirp waveform irradiance of Eq. (18) significantly to increase the chirp waveform duration to $T_{\max}^{p}$. Substituting $T_{\max}^{p}$ to T in Eq. (21) results in $C_{i}=\tau R_{f}$ for both τ ranges, which implies the chirp waveform irradiance must be decreased to a few thousandths or a few ten-thousandths of the irradiance value of Eq. (18). In addition, $T_{\max}^{p}$ values are in the range of seconds in most practical LED/LD-based pulse PA systems, as will be shown later, thus acquiring PA data with the rate of $1/T_{\max}^{p}$ seriously limits the capability of real-time PA measurements. Although $T=T_{\max}^{p}$ is a little bit improper due to these reasons, $T_{\max}^{c}$ and $T_{\max}^{p}$ could be the absolute criteria to straightforwardly determine SNR ratios without considering other conditions. The smallest SNR ratio of Eq. (20) for $T=T_{\max}^{c}$ is increased by multiplying $1/C_{i}$ to Eq. (20) for $T_{\max}^{c}<T$ and ended up to the largest SNR ratio of Eq. (23) for $T=T_{\max}^{p}$.

### 4.3 SNR Ratios for Practical LED/LD-Based PA Systems

Table 1 gives parameters of selected LED/LD-excited pulse train PA studies in the recently published literature,^18^^,^^19^^,^^22^^,^^28^^,^^29^ where the first four cases are reflection-mode PA measurements and the others, Refs. 18 and 29, are the transmission-modes. The bandwidth, Δν of the Ref. 29, in the table has been estimated considering the bandwidth-central frequency ratio in the other Ref. 18. All values for the parameters from Cp to Est. SNR ratio in Table 1 were calculated from the PA measurement condition values directly taken from each reference. The authors in the Ref. 18 estimated the single pulse fluence as 0.1 to $1.6\;\mathrm{mJ}/{\mathrm{cm}}^{2}$, thus we selected the middle value, $0.85\;\mathrm{mJ}/{\mathrm{cm}}^{2}$ as the fluence. For the first case of the Ref. 22, where τ=100  ns, the $C_{\mathrm{pt}}$, $T_{\max}^{c}$, and $T_{\max}^{p}$ values are averaged ones from two different conditions for τ≤100  ns and τ≥100  ns. The term, $T_{f}$ means the time for acquiring a single PA data, such as an A-line, indicated in each reference. For example, $T_{f}=128/4\;\mathrm{KHz}=32\;\mathrm{ms}$ for the PA system of $R_{f}=4\;\mathrm{KHz}$ and averaging 128 pulses.^19^ It can be presumed that the pulse train PA SNR in each reference is sufficiently high with the pulse train duration, $T_{f}$. If this were not the case, the authors of those references would have increased pulse train durations longer than $T_{f}$. Note that the $T_{f}$ values are in the middle of $T_{\max}^{c}$ and $T_{\max}^{p}$ in all references. The $C_{pt}$ values in Eq. (13) for $T=T_{f}$ are also calculated in Table 1, which indicate that the fluence of pulse trains for acquiring a single PA data is typically much less than the ANSI MPE in these practical LED/LD-based pulse train PA systems. It is impossible to know the detail information of the PA imaging configuration in each reference, such as an absorbing object distribution, ultrasound transducer transfer function and so on. Therefore, we estimated γ(τ,Δν) values from the PA imaging configuration assumed in this paper considering actual Δν and $\nu_{c}$ values in Table 1. We expect those estimated γ(τ,Δν) quantities suggest some reasonable ranges for exact γ(τ,Δν) ones. For the most references except the last one, the estimated SNR ratios of Eq. (16) considering estimated γ(τ,Δν) values are smaller than 1. This implies that if matched filtering by chirp waveforms of the duration, $T_{f}$ were considered instead of pulse trains in those LED/LD-based PA systems, the newly obtained SNRs would be higher than SNRs of pulse trains used in those references. For the last reference, where the $T_{f}$ value is much close to $T_{\max}^{p}$ rather than $T_{\max}^{c}$, pulse train PA signals show better SNRs than assumed matched filtered PA signals. However, it can be stated that the SNR difference is quite small compared to the conventionally accepted 20 to 30 dB, which implies the very low fluence per pulse value causes significant reduction of the pulse train SNR, as demonstrated in this paper.

**Table 1:** Parameters from previously published LED/LD-based PA systems.

	19	28	22 First	22 Second	18	29
τ (ns)	70	70	100	200	50	200
$E_{p}$ ($\mathrm{mJ}/{\mathrm{cm}}^{2}$)	0.009	0.044	0.24	0.4	0.85	0.12
$R_{f}$ (KHz)	4	4	1	1	1	40
Δν (MHz)	8.1	5.25	8	8	4.1	2.5 (est.)
$\nu_{c}$ (MHz)	10	7	10	10	3.5	2.25
$T_{f}$ (ms)	32	32	256	256	1	100
$C_{p}$	0.00045	0.0022	0.0121	0.0172	0.0425	0.0052
$C_{pt}(T_{f})$	0.0025	0.0121	0.0785	0.131	0.0043	0.776
$T_{\max}^{c}$ (ms)	1.70	0.211	0.035	0.045	0.0026	0.224
$T_{\max}^{p}$ (s)	95.53	11.51	7.61	3.85	1.41	0.14
$C_{i}(T_{f})$	0.113	0.023	0.0013	0.0015	0.0115	0.0103
Est. γ(τ,Δν)	0.063	0.152	0.0055	$3.5\times 10^{-8}$	0.752	0.238
Est. SNR ratio	0.014	0.133	0.077	$8.2\times 10^{-7}$	0.42	2.92

## 5 Discussion and Conclusion

Bulky pulse lasers can generate high-power pulse trains that are composed of a few tens of nanoseconds pulses with the $C_{p}$ more than a few tenths. If there exists some continuous laser that outputs chirp waveforms whose irradiance is nearly the same as the high pulse power divided by the pulse width, the matched filtered PA signals from chirp waveforms would show higher SNRs than pulse train PA signals even in those bulky pulse lasers. However, the chirp waveform irradiance and duration for this case become too high and short, respectively, so it is almost impossible to practically realize such continuous waveforms. For example, if the matched filtering is alternatively considered instead of the pulse train having a few tens of nanoseconds pulses of $C_{p}∼0.1$, the irradiance and duration of the chirp waveform must be a few hundreds of $\mathrm{KW}/{\mathrm{cm}}^{2}$ and nanoseconds, respectively, at least to outperform the pulse train in terms of an SNR. Very small $C_{p}$ values in LED/LD-based PA systems shift the range of chirp waveform irradiance and duration into the practical region where continuous PA signals are superior to the pulsed ones in terms of an SNR. This concept was theoretically embodied in Eqs. (18), (19), and (22) that are dependent on $C_{p}$. The smaller $C_{p}$ increases both $T_{\max}^{c}$ and $T_{\max}^{p}$ more, which means the waveform duration range showing ${\mathrm{SNR}}_{c}\geq {\mathrm{SNR}}_{pt}$ is more extended. Oppositely, high $C_{p}$ values close to 1 extends the waveform duration range showing ${\mathrm{SNR}}_{c}<{\mathrm{SNR}}_{pt}$, which is similar to the conventional bulky pulse laser situation, where pulse PA signals are dominant in terms of an SNR. Also, the result in this paper indicates that the SNR ratios are different for different $C_{p}$ values even the overall pulse train fluence ($C_{pt}$) is the same. For example, two pulse train waveforms of ($10\;\mu J/{\mathrm{cm}}^{2}$, 1 kHz) and ($1\;\mu J/{\mathrm{cm}}^{2}$, 10 kHz) for the fluence per pulse and pulse repetition rate, respectively, have the same pulse train fluences. However, when matched filtering with chirp waveforms are considered instead of those pulse trains, the rate of SNR enhancement is higher for the latter case than the former. Increasing $C_{p}$ does not always result in the enhancement of SNR ratios, though. If $C_{p}$ of pulse trains is increased only by increasing a single pulse width, τ, the SNR ratio could be dramatically reduced due to the abrupt reduction of γ(τ,Δν) in Eq. (11). We can observe this mechanism from the real LED/LD pulse train PA systems of the Ref. 22, as shown in Table 1.

The duration of chirp waveforms for PA radar is generally in the range of fulfilling the thermal confinement (a few tens of milliseconds), not the stress confinement (a few tens of nanoseconds) for a typical biomedical tissue.^1^^,^^9^^,^^12^^–^^14^ However, it is well known that the validity of Eq. (1) is still fulfilled with such a waveform duration range.^1^ If the chirp waveform duration studied in this paper is longer than the duration range fulfilling the thermal confinement, multiple short chirp waveforms instead of a single long chirp could be considered. It is well known in the radar community that the SNR of matched filtered signals by a chirp waveform is proportional to $\sqrt{T}$ as long as the waveform irradiance is the same.^11^^,^^27^^,^^30^ Let’s make the notations as $T_{\mathrm{ch}}(<T)$ and $T_{o}$ for the waveform duration of multiple chirp waveforms and the interval between those waveforms, respectively. Assuming other parameters are the same, the SNR variation rate of matched filtered PA signals by reducing the chirp waveform duration from T to $T_{ch}$ is $\sqrt{T_{ch}/T}$. Considering a matched filtered PA SNR is increased as the factor of $\sqrt{N_{ch}}$ by averaging $N_{ch}$ number of multiple chirp waveforms, where $N_{\mathrm{ch}}=T/(T_{ch}+T_{o})$, the rate of SNR variation by switching a single chirp waveform to multiple shorter chirp waveforms becomes

$$\sqrt{N_{ch}}\sqrt{\frac{T_{ch}}{T}}=\sqrt{D_{c}},$$ (24)

where $D_{c}=T_{\mathrm{ch}}/(T_{\mathrm{ch}}+T_{o})$, the duty cycle of the multiple chirp waveforms. Consequently, the SNR ratios between pulse train and matched filtered PA signals by multiple chirp waveforms can be acquired by dividing the SNR ratio results in this paper with $\sqrt{D_{c}}$. Practically, $D_{c}$ of multiple chirp waveforms would be quite high because the $T_{ch}$ and $T_{c}$ can be set to a few milliseconds and several tens or hundreds of microseconds, respectively. Even for the case of a 0.5 duty cycle, the additional increasing factor for the SNR ratio is 1.414. Therefore, it can be stated that the effect of considering multiple chirp waveforms for fulfilling the thermal confinement condition on the result in this paper is almost insignificant. Although there has been no stress confinement issue in the previous literature dealing with SNR comparison between PA signals from pulse and continuous waveforms, the stress confinement failure of chirp waveforms might affect the SNR of matched filtered PA signals. Thus, it would be required to conduct the further research addressing this concern with experimental verification.

It is frequent that the pulse fluence of LED/LD-based pulse train PA systems is reduced more than a few thousandths of the ANSI MPE. The result in this paper indicates it would be advantageous in terms of an SNR to operate such very low optical-power PA systems with the matched filtering process with chirp waveforms rather than pulse trains. Investigating SNR ratios from brute-force simulation adopting parameters of the actual LED/LD-based PA systems supports the finding of this paper. In addition, continuously operating LED/LDs circumvents the extra work of fabricating pulse current drivers and the risk of reducing the lifetime of those optical components. We hope the result in this paper extends the research of applying various continuous waveforms already studied in the conventional radar technology to PA systems of limited optical power. This would diversify and expedite the research and development of LED/LD-based, compact, and cost-effective PA systems in the field of biomedical optics.
